# Poor outcome of patients with pulmonary arterial hypertension with insufficient response to phosphodiesterase-5 inhibitors alone or in combination with other specific therapy: a registry-based study

**DOI:** 10.1177/2045894020958557

**Published:** 2020-09-28

**Authors:** Clara Hjalmarsson, Oisin Butler, Roger Hesselstrand, Katsiaryna Holl, Kjell Jansson, Rogier Klok, Göran Rådegran, Stefan Söderberg, Barbro Kjellström

**Affiliations:** 1 Department of Molecular and Clinical Medicine, Sahlgrenska Academy, Gothenburg University, and Department of Cardiology, Sahlgrenska University Hospital, Gothenburg, Sweden; 2 Bayer AG, Berlin, Germany; 3 Department of Clinical Sciences Lund, Rheumatology, Lund University, and Skåne University Hospital, Lund, Sweden; 4 Department of Cardiology and Department of Clinical Physiology, Institution of Medicine and Health Sciences, Linköping University, Linköping Sweden; 5 Merck & Co., Inc., Kenilworth, NJ, USA; 6 Department of Clinical Sciences Lund, Cardiology, The Section for Heart Failure and Valvular Disease, Lund University, and Skåne University Hospital, Lund, Sweden; 7 Department of Public Health and Clinical Medicine, and Heart Centre, Umeå University, Umeå, Sweden; 8 Department of Clinical Sciences Lund, Clinical Physiology, Lund University, and Skåne University Hospital, Lund, Sweden; 9 Cardiology Unit, Department of Medicine, Karolinska Institute, Stockholm, Sweden

**Keywords:** pulmonary hypertension, risk assessment, prognosis

## Abstract

Phosphodiesterase-5 inhibitors are commonly used in pulmonary arterial hypertension but, as suggested by the RESPITE study, phosphodiesterase-5 inhibitor therapy (mono-/combination) does not always have a satisfactory treatment effect. This study aimed to investigate the clinical course of pulmonary arterial hypertension patients not at treatment goal after at least 90 days of treatment with phosphodiesterase-5 inhibitors, alone or in combination with other pulmonary arterial hypertension therapies. The study included 106 incident patients from the Swedish Pulmonary Arterial Hypertension Registry, treated with phosphodiesterase-5 inhibitors for ≥90 days, who were not at a pre-specified treatment goal, i.e. in World Health Organisation functional class III, with 6-min walking distance 165–440 m, and N-terminal prohormone of brain natriuretic peptide >300 ng/L. Changes in World Health Organisation functional class, 6-min walking distance, N-terminal prohormone of brain natriuretic peptide, and risk group between index and follow-up were assessed. Of patients with complete follow-up data, (*n* = 53) 77% were on combination therapy and risk assessment yielded 98% at intermediate risk at index. At follow-up, 11 patients transitioned from World Health Organisation functional class III to World Health Organisation functional class II, the median (Q1; Q3) change in 6-min walking distance was 6 (−30; 42) meters and in N-terminal prohormone of brain natriuretic peptide 47 (−410; 603) ng/L, while 89% remained at an intermediate risk. Of those without complete follow-up data, 11 patients died and 2 underwent lung transplantation. In conclusion, pulmonary arterial hypertension patients treated with phosphodiesterase-5 inhibitors, as single or combination therapy and not achieving the pre-specified treatment goals after ≥90 days have an unfavourable clinical course.

## Introduction

Pulmonary arterial hypertension (PAH) is a rare, chronic disease associated with high mortality, where improvements in treatment strategies over the last decades have led to better symptom control and outcomes.^1–3^ At the 6th World Symposium on Pulmonary Hypertension in Nice in 2018, a modified treatment approach, suggesting initial dual combination therapy with an endothelin receptor antagonist (ERA) and a phosphodiesterase-5 inhibitor (PDE-5i) was recommended for patients at low or intermediate risk.^4^ However, a sizeable proportion of patients with PAH fail to reach or maintain treatment goals with monotherapy and/or combination therapy.^5,6^ Reasons for this may include natural disease progression, lack of drug efficacy, and inadequate compliance or treatment termination due to side effects.

The RESPITE-study (Riociguat Clinical Effects Studied in Patients With Insufficient Treatment Response to PDE-5 inhibitors) investigated replacing PDE-5i therapy with the soluble guanylate cyclase stimulator (s-GCs) riociguat in PAH patients with insufficient response to PDE-5 inhibition.^7^ However, the exploratory nature of the study and the lack of a control arm complicated the interpretation of the study.^7^ Thus, the aim of the present study was to investigate the clinical course of patients with PAH who, in analogy with the RESPITE-study, showed insufficient response after at least 90 days of treatment with a PDE-5i, alone or in combination with other PAH-specific therapy, based on data reported in the Swedish Pulmonary Arterial Hypertension Registry (SPAHR).

## Methods

### Study design and study population

This was an observational descriptive cohort study aimed to investigate the disease course of PAH patients who had not reached a pre-determined treatment goal after 90 days or more on PDE-5i alone or in combination with other PAH-specific therapy. The inclusion criteria of the present study were based on, but not the same as, the selection criteria of the RESPITE study.^7^

Adult, incident patients diagnosed with PAH between January 2008 and July 2019, and registered in SPAHR were assessed for inclusion in the study. Patients were included in the study if they had been treated with PDE-5i for ≥90 days and if they were confirmed not to be at a pre-specified treatment goal, which was defined as World Health Organisation functional class (WHO FC) III, 6-min walking distance (6MWD) 165–440 m, and N-terminal prohormone of brain natriuretic peptide (NT-proBNP) >300 ng/L.^7^ Patients were excluded if they did not have measurements available from all three variables (i.e. WHO FC, 6MWD, and NT-proBNP) or if values were out of the pre-specified ranges at the first follow-up after being treated with PDE-5i ≥90 days (index date).

The index date was defined as the first registered visit after the diagnosis where the patient met the inclusion criteria. The first available visit that occurred in a window of 6–18 months after the index date was defined as follow-up. Clinical parameters and information on PAH-specific treatment at index date and follow-up were extracted from the SPAHR.

Risk assessment used the SPAHR risk calculating model^5^ based on the risk assessment instrument from the European Society of Cardiology and European Respiratory Society ESC/ERS guidelines.^2,8^ Variables available included WHO FC, 6MWD, NT-proBNP, right atrial area, mean right atrial pressure, pericardial effusion, cardiac index, and mixed venous oxygen saturation. Each variable was graded from 1 to 3 where 1 = ‘Low risk’, 2 = ‘Intermediate risk’, and 3 = ‘High risk’, and the sum of all grades was divided by the number of available variables for each patient rendering a mean grade. The mean grade was rounded off to the nearest integer, which was then used to define the patient’s risk group.

Creatinine levels were used to estimate glomerular filtration rate according to the Modification of Diet in Renal Disease equation.^9^ Hemodynamic measurements and comorbidities were only available at time of diagnosis.

### End-points

Changes in WHO FC, 6MWD, NT-proBNP, and risk group between index and follow-up were assessed in all patients who fulfilled the inclusion criteria and had complete data at the follow-up visit.

### Swedish PAH registry and ethical approval

Data recorded in SPAHR^3,10^ were used for the present study. SPAHR was started in 2008 and constitutes an open continuous register with the intention to include all Swedish patients diagnosed with PAH according to the Dana Point or Nice classification. The PAH diagnosis was made by right heart catheterization according to the ESC/ERS guidelines for the diagnosis and treatment of pulmonary hypertension effective at the time of diagnosis.^2,8^ All Swedish PAH centres report data to SPAHR, thereby enabling a high national coverage of >90%.^3,10^ SPAHR was approved by the National Board of Health and Welfare and the Swedish Data Protection Authority. All patients were informed about their participation in SPAHR and had the right to decline. The present study complies with the Declaration of Helsinki and was approved by the national ethics committee in Sweden (Dnr. 2019-04210).

### Statistical methods

Descriptive statistics was used to characterize the data. Changes in median values between index and follow-up were tested by signed rank test; *p* values <0.05 were regarded as significant. All analyses were carried out by use of the SAS statistical software (The SAS system for Windows 9.4, SAS Institute Inc., Cary, NC, USA).

## Results

### Patient characteristics

**Fig. 1.**
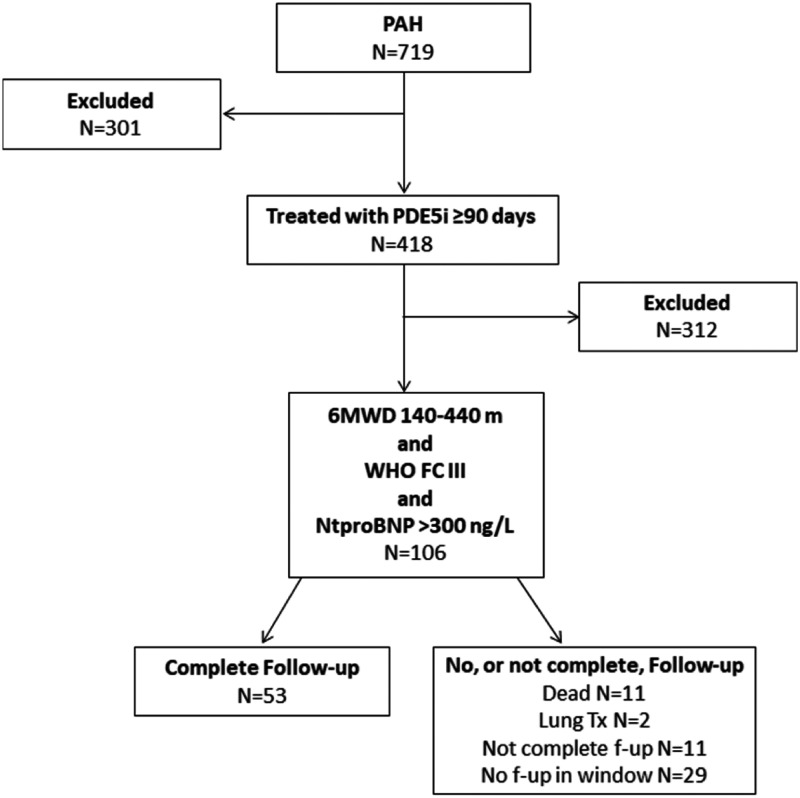
Flowchart of patients who met inclusion/exclusion criteria for the study population.

Between January 2008 and July 2019, 719 incident patients with PAH were reported to SPAHR (Fig. 1). Of those, 418 (58%) patients had been treated with PDE-5i for at least 90 days, whereof 106 (25%) patients met the inclusion criteria. Three hundred and twelve patients did not meet the inclusion criteria, i.e. they did not have measurements available from all three variables (WHO FC, 6MWD, and NT-proBNP) at the first follow-up after being treated with PDE-5i ≥90 days (index date) or the measurements from these three variables were outside the ranges for inclusion.

The demographic, clinical, and hemodynamic characteristics of the whole SPAHR cohort were similar to the sub-group (*n* = 418) treated with PDE-5i, for details see Supplemental Table 1. Compared to the 312 patients who were excluded, the 106 patients included in the study were older, had a higher comorbidity burden, in particular diabetes mellitus and atrial fibrillation and fewer were at low risk, for details see Supplemental Table 2.

**Table 1.** Patient characteristics at index date by available follow-up data (*N* = 106).

	With follow-up	Without follow-up and alive	Without follow-up and dead/transplanted
	(*n* = 53)	(*n* = 40)	(*n* = 13)
Time from diagnosis to Index date (days)	264 (166–482)	245 (156–407)	219 (168–287)
Age (years)	69 (65–75)	69 (63–76)	70 (58–77)
Gender, female	32 (60)	26 (65)	8 (62)
WHO-FC (III)	53 (100)	40 (100)	13 (100)
BMI (kg/m^2^)	26 (24–28)	26 (22–29)	25 (20–28)
6MWD (m)	274 (210–335)	289 (216–355)	300 (219–335)
eGFR (ml/min/1.73 m^2^)	61 (50–76)	54 (43–70)	60 (48–72)
Hb (g/L)	131 (118–150)	120 (113–147)	137 (119)
NTproBNP (ng/L)	1050 (653–2598)	1454 (744–2708)	3065 (1199–3645)
PAH-classification			
IPAH/HPAH	31 (58)	21 (53)	7 (54)
APAH-CTD	16 (30)	12 (30)	4 (31)
APAH-CHD	3 (6)	6 (15)	0
APAH-Other	3 (6)	1 (3)	2 (15)
Comorbidities^a^			
Hypertension	24 (49)	17 (46)	6 (50)
Diabetes Mellitus	14 (29)	8 (22)	4 (33)
Atrial fibrillation	11 (23)	10 (27)	3 (25)
Ischemic heart disease	9 (18)	10 (27)	1 (8)
Ischemic stroke	5 (10)	3 (8)	0
No of comorbidities^a^			
0 comorbidities	15 (28)	11 (28)	5 (38)
1 comorbidities	12 (23)	9 (23)	4 (31)
≥2 comorbidities	26 (49)	19 (49)	4 (31)
Invasive hemodynamics^a^			
MRAP (mm Hg)	8 (4–11)	8 (5–11)	5 (2–10)
MPAP (mm Hg)	47 (40–55)	39 (32–52)	40 (37–43)
PAWP (mm Hg)	10 (7–13)	8 (5–13)	9 (5–12)
CI (L/min/m^2^)	2.3 (1.9–2.6)	2.3 (1.8–2.8)	2.3 (2.0–2.9)
PVR (wood units)	8.5 (6.0–11.9)	7.4 (5.4–10.2)	7.9 (4.6–10.0)
SvO2 (%)	60 (54–65)	62 (56–68)	64 (56–68)
SaO2 (%)	92 (85–94)	91 (88–94)	93 (90–95)
Echocardiography			
RA area (cm^2^)	25 (21–30)	23 (21–27)	22 (20–28)
Pericardial effusion	5 (12)	5 (16)	0
PAH-targeted therapy			
PDEI	12 (22)	15 (37)	5 (39)
PDEI + ERA	37 (70)	19 (48)	7 (54)
PDEI + PRO	2 (4)	–	–
PDEI + ERA + PRO	2 (4)	6 (15)	1 (7)
Supportive therapy			
Anticoagulants	34 (64)	23 (58)	7 (54)
Diuretics	44 (83)	37 (93)	11 (85)
Supplemental oxygen	15 (28)	12 (30)	3 (23)
Risk group^5^			
Low risk	0	3 (7)	1 (8)
Intermediate risk	52 (98)	33 (83)	12 (92)
High risk	1 (2)	4 (10)	0

Data presented as median (Q1–Q3) or *n* (%). WHO-FC: functional class; BMI: body mass index; 6MWD: 6-min walked distance; eGFR: estimated glomerular filtration rate; Hb: hemoglobin; NTproBNP: N-terminal pro brain natriuretic peptide; PAH: pulmonary arterial hypertension; IPAH: idiopathic pulmonary arterial hypertension; FPAH: familiar pulmonary arterial hypertension; APAH: associated pulmonary arterial hypertension; CTD: connective tissue disease; CHD: congenital heart disease; MRAP: mean right atrial pressure; MPAP: mean pulmonary artery pressure; PAWP: pulmonary artery wedge pressure; CI: cardiac index; PVR: pulmonary vascular resistance; SvO2: mixed venous oxygen saturation; SaO2: arterial oxygen saturation; RA: right atrium; PDEI: phosphodiesterase-5 inhibitors; ERA: endothelin receptor antagonist; PRO: prostacyclin or prostacyclin derivative.

a Registered at the time of diagnosis. Four patients did not have complete data on comorbidities.

**Table 2.** Characteristics of the study population at index and follow-up (*n* = 53).

	Index *n*	Index	Follow-up *n*	Follow-up	Change	*P* value^a^
Age (years)	53	69 (65–75)	53			
BMI (kg/m^2^)	44	26 (24–28)	43	26 (22–29)	0 (−1 – 0)	0.2432
WHO-FC I/II/III/IV, %	53	0/0/100/0	53	0/21/77/2		
6MWD (m)	53	274 (210–335)	53	285 (206–348)	6 (−30 – 42)	0.4776
eGFR (ml/min/1.73 m^2^)	53	61 (50–76)	50	59 (45–75)	−3 (−10 − 5)	0.0811
Hb (g/L)	52	131 (118–150)	51	136 (119–156)	1 (−7 – 10)	0.3174
NTproBNP (ng/L)	53	1050 (653–2598)	53	1310 (574–2690)	47 (−410 – 603)	0.4606
Echocardiography						
RA area (cm^2^)	26	25 (17–29)	20	27 (21–32)	0 (−4 – 3)	0.8066
Pericardial effusion	19	2 (6)	19	1 (4)	1 (4)	
Supportive therapy						
Anticoagulants	53	34 (64)	53	35 (66)	3	
Diuretics	53	44 (83)	53	41 (77)	5	
Supplemental oxygen	53	15 (28)	53	18 (34)	3	

Data are presented as median (1st–3rd quartile) or *n*(%). WHO-FC: functional class; BMI: body mass index; 6MWD: 6-min walking distance; eGFR: estimated glomerular filtration rate; Hb: hemoglobin; NTproBNP: N-terminal pro brain natriuretic peptide; RA: right atrium.

a Signed Ranked test.

Of the 106 included patients, 53 patients had complete data at follow-up (Fig. 1) and were included in the final analyses. Among the 53 patients with missing data at follow-up, 2 patients underwent lung transplantation and 11 patients had died during the follow-up time, while 11 patients did not have complete data at follow-up and 29 did not have a recorded visit within the pre-specified follow-up window (Fig. 1).

### Index

Patients with complete follow-up data had median (1st–3rd quartile) time from diagnosis to the index visit of 8.7 (5.4–15.8) months, while the median time that elapsed between index and follow-up was 8.9 (6.7–11.7) months. Median time from start of PDE-5i to index visit was 5.3 (3.8–9.0) months. The median age at index date was 69 (65–75) years, and 60% were women (Table 1). More than half of the study group had idiopathic PAH /heritable PAH and a third had PAH associated to connective tissue disease (CTD-APAH). Patients were slightly overweight, had a low 6MWD, 274 (210–335) m, and the NT-proBNP was 1050 (653–2598) ng/L.

**Fig. 2.**
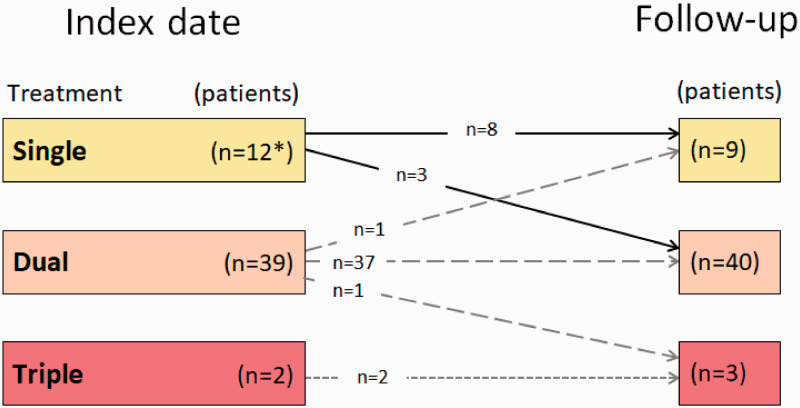
Change in treatment patterns between index and follow-up visits. One patient with single therapy at baseline terminated treatment during the follow-up period.

Risk assessment yielded 98% at intermediate risk. A majority of patients were on combination therapy including a PDE-5i (77%), while 23% were treated with PDE-5i monotherapy (Fig. 2). A considerable proportion of patients received supportive therapy with anticoagulants, diuretics, or supplemental oxygen (Table 1).

Demographics and the clinical characteristics were similar for patients with and those without complete follow-up data (Table 1).

### Follow-up

**Fig. 3.**
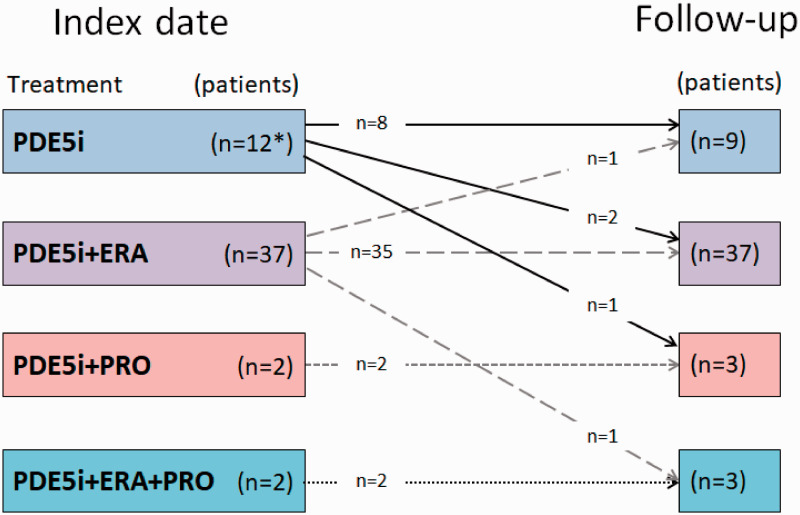
Change in specific PAH-drugs between index and follow-up visits. One patient on PDE-5i therapy at baseline terminated treatment during the follow-up period.

Most of the patients continued with the same combination therapy at follow-up as at index (Fig. 3). Three patients on monotherapy and one patient on dual therapy added one drug, while one patient transitioned from dual to single treatment and one patient terminated the treatment during follow-up.

**Fig. 4.**
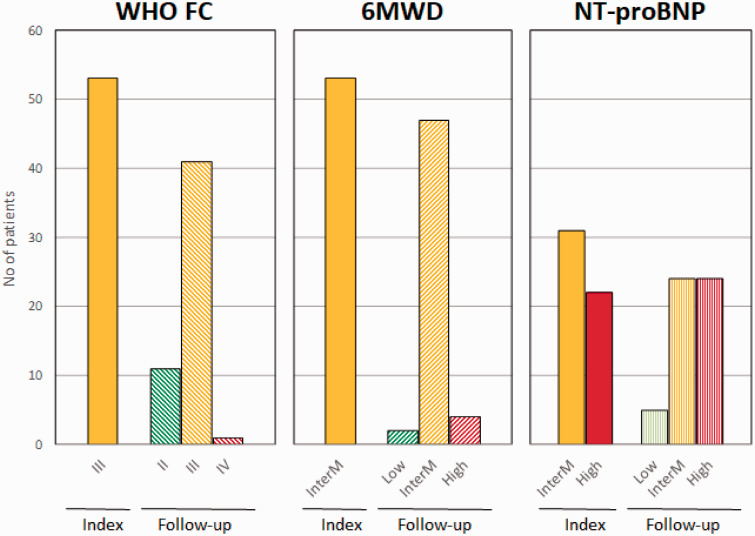
Change in number of patients reaching the treatment goals of WHO functional class (WHO FC), 6 min walked distance (6MWD; low ≤ 165 m, intermediate = 165–440 m, high ≥ 440 m) and NT-proBNP (low ≤ 300 ng/L, intermediate = 300–1440 ng/L, high ≥ 1440 ng/L) between index and follow-up.

At the follow-up visit, the clinical characteristics of most patients remained unchanged (Table 2). Eleven patients (21%) transitioned from WHO FC III to WHO FC II. Few patients improved in regards to 6MWD and NT-proBNP (Fig. 4). The median change in 6MWD was 6 (−30 to 42; *p* = 0.478) meters and in NT-proBNP 47 (−410 to 603; *p* = 0.461) ng/L.

**Fig. 5.**
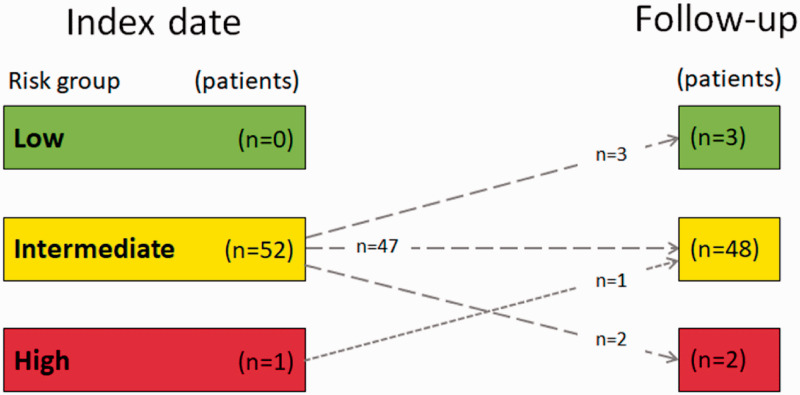
Change in risk assessment score from index date to follow-up visit.

In regards to risk assessment, most of the patients (89%) remained at an intermediate risk while four patients (7%) improved to a lower risk and two patients (4%) deteriorated, reaching high risk (Fig. 5).

## Discussion

This study investigated the clinical course of patients with PAH in a selected register population treated with a stable dose of a PDE-5i for ≥90 days and not at treatment goal.

The main finding of the present study is that, despite the fact that 77% of the patients were treated with combination PAH-therapy at index date, very few patients showed a clinical improvement between index and follow-up. This is not surprising, as only four patients had an escalated treatment, three from single to dual, and one from dual to triple treatment, at the follow-up visit. Other findings were that a noteworthy number of patients died or received lung transplantation during the study period, that most of the patients had an intermediate risk profile at diagnosis, which remained unchanged during the follow-up period and, that the population was generally old and had a pronounced comorbidity burden.

It has been reported that some patients receiving PDE-5i therapy deteriorate clinically over time, suggesting the possibility of optimizing the cyclic guanosine monophosphate (cGMP) pathway with another agent.^11^ The RESPITE-study investigated the potential clinical improvement in patients not at treatment goal by switching therapies from PDE-5i to the s-GC stimulator riociguat. More than half of the patients improved their WHO FC and in addition, both 6MWD and NT-proBNP improved significantly. However, the investigators acknowledged that one of the limitations of the study was the absence of a control group.^7^ An ongoing, prospective, randomized clinical trial, the REPLACE-study (Riociguat rEplacing PDE-5i Therapy evaLuated Against Continued PDE-5i therapy) will help to elucidate the clinical effect of switching patients not at treatment goal from a PDE-5i to riociguat (clinicaltrials.gov identifier NCT02891850).

In the meantime, our data, describing a real-life patient cohort, might shed light on the clinical course of patients on PDE-5i who, in analogy to the RESPITE-inclusion criteria, were not at a pre-specified treatment goal at study start. In the present study, patients treated with riociguat were excluded and changes in treatment, WHO FC, 6MWD, NT-proBNP, and risk group between index date and the first consecutive follow-up 6–18 months later were investigated.

The treatment patterns seen at study start remained similar to those at the follow-up, and the outcome measures of WHO FC, 6MWD, and NT-proBNP did not change significantly over the course of the study. In terms of risk assessment, only a fraction of the patients included in the present study improved to a low risk at follow-up, while most remained in the intermediate risk group. While combination therapy was not uncommon, three quarters of the patients were treated with two or more PAH-drugs, this did not seem to be enough to improve the outcome of this progressive disease. The patients who fulfilled the inclusion criteria were older and had a more pronounced comorbidity burden compared to patients not included in the study. It is possible that age and comorbidity influenced both the likelihood that patients received treatment escalation and the likelihood of response to escalation, as age as well as comorbidities such as atrial fibrillation, have been shown to impact outcome in PAH.^12–15^

Moreover, a third of the patients were diagnosed with CTD-APAH. While this clinical subgroup has been shown to benefit from treatment with PDE-5i, single or in combination with ERA, compared to ERA alone,^16^ in general they have a poorer prognosis than IPAH.^17,18^ This, together with advanced age and comorbidity, might also have contributed to the high mortality rate during the follow-up period,^12,13^ and the rather high proportion of patients not having complete follow-up data during the study period.

Interestingly, although the study group was quite homogenous in respect to demographics and functional characteristics at index, as well as comorbidity and hemodynamic characteristics at time of diagnosis, some patients died or received lung transplantation during the follow-up period while other remained stable. This might be due to individual patient characteristics, which discriminate between responders and non-responders.^19^ However, whether differences in outcomes are due to better individual treatment response, superior treatment adherence of some patients, different disease characteristics or other factors in the present study, is difficult to know. With a retrospective study design, it is not possible to assess if a more aggressive treatment or another combination approach might have led to different results. Nevertheless, the results undeniably indicate the poor outcome of this selected group of patients with PAH. Though most of them were assessed to be at an intermediate risk, their mortality rate was in parity to the one expected for patients in the high risk category. This highlights the need of refined methods for clinical assessment and individualized treatment strategies.

### Study limitations

SPAHR is a register based in Sweden, which means that the population is Sweden-specific. This might impact the generalizability of the study outcomes toward other countries. A considerable number of patients had visits without complete follow-up data while some had no visit registered within the follow-up window. One may speculate that some of these patients had milder disease and, thus, more seldom follow-up visits. Nonetheless, the baseline characteristics of the patients who did not have a follow-up were not different from those of the patients with complete follow-up data. Finally, in the present study, few patients underwent right heart catheterisation at the study index date and/or follow-up, and therefore, no hemodynamic data were included in the analysis.

The study is prone to common standard limitations of a registry-based descriptive study, such as lack of a standardized study protocol, handling missing data and the possibility of selection bias with respect to follow-ups. Data entry into SPAHR is manual and introduces a possible error that could affect data quality. However, monitoring and validation of register data in relation to data sources (the patient files) was performed 2015, and the results showed that more than 95% of all key variables were entered correctly.

## Conclusion

The findings point toward unsatisfactory treatment effects in this selected group of patients. Several reasons could play a role for this outcome, among them demographic and disease-related characteristics of the study population, as well as treatment patterns. The results highlight the need for refined risk assessment and improved clinical strategies, such as earlier transitioning or escalation of PAH-targeted therapy.

## Supplemental Material

sj-pdf-1-pul-10.1177_2045894020958557 - Supplemental material for Poor outcome of patients with pulmonary arterial hypertension with insufficient response to phosphodiesterase-5 inhibitors alone or in combination with other specific therapy: a registry-based study Click here for additional data file. Supplemental material, sj-pdf-1-pul-10.1177_2045894020958557 for Poor outcome of patients with pulmonary arterial hypertension with insufficient response to phosphodiesterase-5 inhibitors alone or in combination with other specific therapy: a registry-based study by Clara Hjalmarsson, Oisin Butler, Roger Hesselstrand, Katsiaryna Holl, Kjell Jansson, Rogier Klok, Göran Rådegran, Stefan Söderberg and Barbro Kjellström in Pulmonary Circulation
