# Supplementary material for: Poor outcome of patients with pulmonary arterial hypertension with insufficient response to phosphodiesterase-5 inhibitors alone or in combination with other specific therapy: a registry-based study
Source: Pulm Circ. 2020 Sep 28;10(3):2045894020958557. doi: 10.1177/2045894020958557 (PMC7534092; doi:10.1177/2045894020958557)
Supplement: sj-pdf-1-pul-10.1177_2045894020958557 - Supplemental material for Poor outcome of patients with pulmonary arterial hypertension with insufficient response to phosphodiesterase-5 inhibitors alone or in combination with other specific therapy: a registry-based study [file sj-pdf-1-pul-10.1177_2045894020958557.pdf]

Supplemental Table 1. Characteristics at time of diagnosis for all patients and patients on PDE5i ≥90 days. Data presented as median (1st-3rd quartile) or n (%).

	All patients (n=719)	Patients on PDE5i >90 days (n=418)
Age (years)	67 (53-73)	67 (51-73)
Gender, female	458 (64)	262 (63)
PAH classification		
IPAH/HPAH	356 (50)	231 (55)
APAH-CTD	227 (32)	124 (30)
APAH-CHD	77 (11)	27 (7)
APAH-Other	59 (8)	36 (9)
WHO-FC (% I/II/III/IV*)	1/18/71/10	1/19/73/8
BMI (kg/m^2)	25 (23-29)	26 (23-29)
6MWD (m)	285 (188-408)	300 (195-408)
DLCO % predicted	44 (32-58)	45 (33-60)
eGFR (ml/min/1.73 m^2)	68 (54-85)	67 (52-86)
Hb (g/L)	143 (128-156)	143 (130-157)
NTproBNP (ng/L)	1430 (440-3350)	1445 (524-3220)
**Right heart catheterization**		
MRAP (mm Hg)	7 (4-11)	6 (4-10)
MPAP (mm Hg)	45 (37-53)	45 (37-53)
PAWP (mm Hg)	8 (6-11)	8 (6-11)
CI (L/min/m2)	2.4 (1.9-2.8)	2.4 (1.9-2.8)
PVR (Wood units)	8.5 (5.9-11.6)	8.5 (6.1-11.6)
SvO2 (%)	62 (55-69)	62 (56-68)
SaO2 (%)	92 (88-95)	92 (88-95)
**Echocardiography**		
RA area (cm2)	22 (19-27)	22 (19-27)
Pericardial effusion	74 (13)	42 (12)
**Comorbidities**		
Hypertension	272 (42)	168 (43)
Diabetes Mellitus	126 (19)	74 (19)
Atrial fibrillation	99 (15)	58 (15)
Ischemic stroke	37 (6)	21 (5)
Ischemic heart disease	93 (15)	53 (14)
Obesity	132 (20)	78 (20)
Renal dysfunction	24 (3)	17 (4)

**No of comorbidities**		
0 comorbidities	282 (40)	162 (39)
1 comorbidities	193 (27)	109 (26)
≥2 comorbidities	236 (33)	144 (35)
**Risk group** [5]		
Low risk	153 (21)	84 (20)
Intermediate risk	492 (69)	295 (71)
High risk	71 (10)	39 (9)
**PAH-targeted therapy**		
ERA	467 (65)	250 (60)
PDE5i	344 (48)	263 (63)
PRO	38 (5)	19 (5)
No treatment	93 (13)	43 (10)
**PAH-targeted therapy**		
Single	418 (67)	228 (61)
Dual	193 (31)	137 (36)
Triple	15 (2)	10 (3)
**Supportive therapy**		
Anticoagulants	354 (51)	213 (51)
Diuretics	444 (62)	264 (63)
Supplemental oxygen	179 (25)	94 (23)

PAH=pulmonary arterial hypertension, IPAH=idiopathic PAH, FPAH=familiar PAH, APAH=associated PAH, CTD=connective tissue disease, CHD=congenital heart disease, WHO-FC=functional class (*presented only as percent), BMI=body mass index, 6MWD=6-minute walked distance, DLCO= diffusing capacity of lung for carbon monoxide, eGFR=estimated glomerular filtration rate, Hb=hemoglobin, NTproBNP= N-terminal pro brain natriuretic peptide, MRAP=mean right atrial pressure, MPAP=mean pulmonary artery pressure, PAWP= pulmonary artery wedge pressure, CI=cardiac index, PVR=pulmonary vascular resistance, RA=right atrium, ERA= endothelin receptor antagonist, PDE-5i=phosphodiesterase-5 inhibitor, PRO=prostacyclin

Supplemental Table 2. Characteristics at time of diagnosis for patients on PDE5i ≥90 days by meeting/not meeting the inclusion criteria. Data presented as median (1st-3rd quartile) or n (%).

	Patients on PDE5i ≥90 days Meeting inclusion criteria (n=106)	Patients on PDE5i ≥90 days Not meeting inclusion criteria (n=312)
Age (years)	69 (63-75)	65 (48-72)
Gender, female	66 (62)	196 (63)
PAH classification		
IPAH/HPAH	59 (56)	172 (55)
APAH-CTD	32 (30)	92 (30)
APAH-CHD	9 (9)	18 (6)
APAH-Other	6 (6)	30 (10)
WHO-FC (% I/II/III/IV*)	-/11/83/6	1/22/69/8
BMI (kg/m^2)	26 (24-29)	26 (23-29)
6MWD (m)	251 (175-355)	325 (210-424)
DLCO % predicted	43 (32-53)	46 (34-65)
eGFR (ml/min/1.73 m^2)	60 (48-76)	68 (54-90)
Hb (g/L)	142 (127-158)	143 (130-157)
NTproBNP (ng/L)	1885 (890-3711)	1281 (353-3110)
**Right heart catheterization**		
MRAP (mm Hg)	7 (4-11)	6 (4-10)
MPAP (mm Hg)	43 (35-52)	46 (38-54)
PAWP (mm Hg)	9 (6-13)	8 (6-11)
CI (L/min/m2)	2.3 (1.9-2.7)	2.4 (2.0-2.8)
PVR (Wood units)	7.7 (5.7-10.6)	8.7 (6.2-11.8)
SvO2 (%)	61 (55-67)	62 (56-69)
SaO2 (%)	91 (88-94)	92 (89-95)
**Echocardiography**		
RA area (cm2)	24 (21-28)	22 (18-26)
Pericardial effusion	10 (12)	32 (13)
**Comorbidities**		
Hypertension	47 (48)	121 (42)
Diabetes Mellitus	26 (27)	48 (16)
Atrial fibrillation	24 (25)	34 (12)
Ischemic stroke	8 (8)	13 (5)
Ischemic heart disease	20 (20)	33 (11)
Obesity	21 (20)	57 (19)

Renal dysfunction	4 (4)	13 (4)
**No of comorbidities**		
0 comorbidities	31 (30)	131 (42)
1 comorbidities	25 (24)	84 (27)
≥2 comorbidities	49 (47)	95 (31)
**Risk group** [5]		
Low risk	10 (9)	74 (24)
Intermediate risk	88 (83)	207 (66)
High risk	8 (8)	31 (10)
**PAH-targeted therapy**		
ERA	63 (59)	187 (60)
PDE5i	58 (55)	205 (66)
PRO	6 (6)	13 (4)
No treatment	13 (12)	30 (10)
**PAH-targeted therapy**		
Single	61 (66)	167 (59)
Dual	30 (32)	107 (38)
Triple	2 (2)	8 (3)
**Supportive therapy**		
Anticoagulants	57 (54)	156 (50)
Diuretics	73 (69)	191 (61)
Supplemental oxygen	23 (22)	71 (23)

PAH=pulmonary arterial hypertension, IPAH=idiopathic PAH, FPAH=familiar PAH, APAH=associated PAH, CTD=connective tissue disease, CHD=congenital heart disease, WHO-FC=functional class (*presented only as percent), BMI=body mass index, 6MWD=6-minute walked distance, DLCO= diffusing capacity of lung for carbon monoxide, eGFR=estimated glomerular filtration rate, Hb=hemoglobin, NTproBNP= N-terminal pro brain natriuretic peptide, MRAP=mean right atrial pressure, MPAP=mean pulmonary artery pressure, PAWP= pulmonary artery wedge pressure, CI=cardiac index, PVR=pulmonary vascular resistance, RA=right atrium, ERA= endothelin receptor antagonist, PDE-5i=phosphodiesterase-5 inhibitor, PRO=prostacyclin

Supplemental Table 3. Characteristics at first follow-up (index date) on PDE5i ≥90 days by those meeting the inclusion criteria and having complete follow-up data and those not meeting the inclusion criteria. Data presented as median (1st-3rd quartile) or n (%).

	Patients on PDE5i ≥90 days Included (n=53)	Patients on PDE5i ≥90 days Not meeting inclusion criteria (n=312)
Days since diagnosis	264 (166-482)	243 (146-460)
Age (years)	69 (65-75)	66 (49-73)
Gender, female	32 (60)	196 (63)
PAH classification		
IPAH/HPAH	31 (58)	172 (55)
APAH-CTD	16 (30)	92 (30)
APAH-CHD	3 (6)	18 (6)
APAH-Other	3 (6)	30 (10)
WHO-FC (I/II/III/IV) %	(-/-/100/-)	(6/44/42/8)
BMI (kg/m^2)	26 (24-28)	25 (23-28)
6MWD (m)	274 (210-335)	382 (255-510)
eGFR (ml/min/1.73 m^2)	61 (50-76)	68 (52-89)
Hb (g/L)	131 (118-150)	136 (122-148)
NTproBNP (ng/L)	1050 (653-2598)	476 (178-1744)
**Echocardiography**		
RA area (cm2)	25 (21-30)	20 (18-24)
Pericardial effusion	5 (12)	14 (8)
**Risk group** [5]		
Low risk	0	118 (38)
Intermediate risk	52 (98)	158 (51)
High risk	1 (2)	33 (11)

PAH=pulmonary arterial hypertension, IPAH=idiopathic PAH, FPAH=familiar PAH, APAH=associated PAH, CTD=connective tissue disease, CHD=congenital heart disease, WHO-FC=functional class, BMI=body mass index, 6MWD=6-minute walked distance, eGFR=estimated glomerular filtration rate, Hb=hemoglobin, NTproBNP= N-terminal pro brain natriuretic peptide, RA=right atrium
